# Bile Acids as Key Mediators of the Gut Microbiota–Immune Axis: Potential Biomarker and Therapeutic Perspectives

**DOI:** 10.1002/biof.70078

**Published:** 2026-01-26

**Authors:** Simone Baldi, Margherita Turrini, Francesco Cei, Marta Menicatti, Gianluca Bartolucci, Amedeo Amedei

**Affiliations:** ^1^ Department of Experimental and Clinical Medicine University of Florence Florence Italy; ^2^ Department of Neurosciences, Psychology, Drug Research and Child Health University of Florence Florence Italy; ^3^ Network of Immunity in Infection, Malignancy and Autoimmunity (NIIMA) Universal Scientific Education and Research Network (USERN) Florence Italy

## Abstract

Bile acids (BAs), long recognized for their role in lipid digestion, have recently emerged as key signaling molecules at the interface of host metabolism, immunity, and gut microbiota (GM). BAs are synthesized in hepatocytes and subsequently extensively modified by microbial enzymes in the gut, producing a diverse and dynamic pool that strongly shapes the GM–immune axis. Through activation of receptors such as the Farnesoid X receptor and the G protein–coupled receptor TGR5, BAs regulate inflammation, metabolic pathways, and intestinal immune homeostasis, particularly influencing the balance between regulatory T cells and pro‐inflammatory Th17 cells. Microbial transformations, primarily deconjugation and 7α‐dehydroxylation, further diversify BA species, modulating receptor affinities and immunoregulatory functions. Dysbiosis‐associated alterations in these processes contribute to the pathogenesis of inflammatory disorders, including inflammatory bowel disease (IBD). Consequently, BAs are increasingly recognized as promising biomarkers for monitoring disease activity and predicting therapeutic response, although validation in standardized, prospective cohorts remains necessary. Recent advances in high‐resolution analytical techniques, notably high‐ and ultra‐performance liquid chromatography coupled with tandem mass spectrometry (HPLC– and UPLC–MS/MS), have enabled precise, high‐throughput quantification of BA species in serum and fecal samples. These methods both deepen mechanistic understanding of BA‐mediated immunomodulation and support the development of GM‐ and BA‐targeted therapies. This review emphasizes the central role of BAs in GM–immune axis regulation, delineates their complex interplay with host and microbial factors, and surveys evolving analytical strategies that facilitate their study in health and disease.

## Graphical abstract

Primary bile acids synthesized in the liver are transformed by gut microbes into secondary bile acids that engage receptors such as FXR and TGR5, thereby modulating immune function and metabolic homeostasis. Dysregulation of these signaling pathways contributes to MASH, NAFLD, IBD, and neurodegenerative disorders, underscoring their relevance as therapeutic targets.

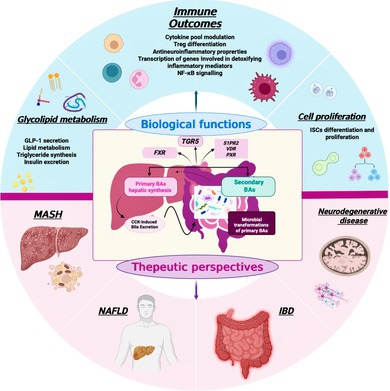

## 1 Introduction

Bile acids (BAs) are essential constituents of bile and, as emulsifying agents, play a crucial role in the digestion and absorption of dietary lipids and fat‐soluble vitamins. In addition to their digestive functions, BAs act as bioactive signaling molecules that modulate a wide range of physiological processes, including lipid and glucose metabolism, energy homeostasis and brain function [1]. Specifically, through interactions with several receptors, most notably the Farnesoid X receptor (FXR) and the Takeda G protein‐coupled receptor 5 (TGR5), BAs contribute to the regulation of inflammatory pathways and immune responses [2, 3, 4]. Dysregulated BA accumulation can therefore exacerbate pathological conditions such as cholestasis and inflammatory bowel disease (IBD), including Crohn's disease (CD) and ulcerative colitis (UC) [5, 6].

Focusing on references selected for their relevance and scientific impact, with particular emphasis on high‐quality reviews and primary clinical studies, this review provides a comprehensive overview of the roles of BAs in both physiological and pathological contexts.

Although the direct causal relationship between BA receptor activation and immunomodulatory effects remains under investigation, emerging research aims to elucidate the mechanisms by which distinct BA species selectively activate FXR, TGR5, and other receptors, thereby influencing inflammatory outcomes [7]. Furthermore, the dynamic crosstalk between BAs and the gut microbiota (GM) adds an additional layer of complexity to this regulatory network. In particular, secondary BAs produced by intestinal bacteria often differ in receptor‐binding affinity and functional effects, opening avenues for innovative therapeutic strategies that target BA signaling pathways [8].

For these reasons, clinical research has increasingly recognized the relevance of BAs, proposing their concentrations as potential biomarkers for pathological conditions, especially inflammation‐mediated disorders [9]. Consequently, specialized analytical methods have been developed in recent years to detect and quantify BAs in human biological samples, including fecal and serum matrices. However, BA analysis presents significant challenges due to (i) the large number of structurally similar or isomeric species, (ii) their low volatility and (iii) their typically low concentrations in biological matrices. As a result, highly sensitive and specific analytical methods are required. Traditional approaches such as gas chromatography–mass spectrometry (GC–MS) have largely been replaced by high‐performance liquid chromatography–tandem mass spectrometry (HPLC–MS/MS) [10].

Hence, this review highlights the interplay between BAs and the host GM–immunity axis and summarizes recent advances in analytical methodologies for the detection and quantification of BAs in human biological samples, underscoring their potential as biomarkers and therapeutic targets.

## 2 Regulation of the BA Pool by the Liver and GM

### 2.1 Biosynthesis of Primary BAs and Human Enterohepatic Circulation

BAs are sterol derivatives characterized by a hydrophobic cyclopentanephenanthrene core bearing a terminal carboxyl group (–COOH) on the side chain, typically at C‐24. BA synthesis occurs primarily in the liver through two main pathways—the classical (or neutral) pathway and the alternative (or acidic) pathway—both of which are conserved in humans and mice. In addition, two auxiliary pathways are noteworthy: the 25‐hydroxylase pathway, mediated by CYP27A1and primarily active in hepatocytes, and the 24‐hydroxylase pathway, mediated by CYP46A1, which is mainly expressed in neurons of the cerebral cortex and hippocampus.

**FIGURE 1:**
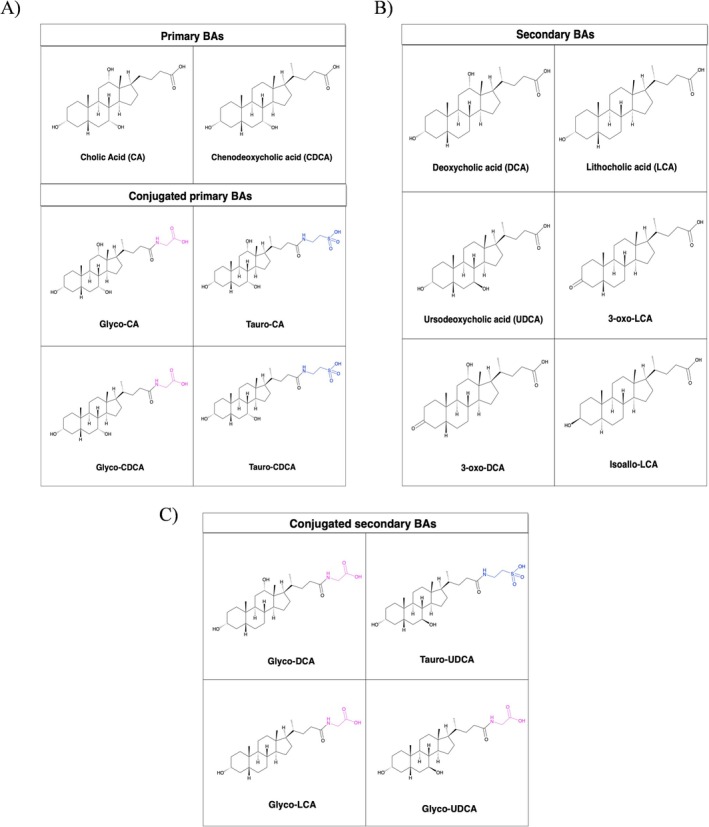
Main chemical structures of (A) primary and conjugated primary BAs, (B) secondary BAs and (C) conjugated secondary BAs. The regions highlighted in pink and blue represent glycine and taurine, respectively. The structural diversity and conjugation patterns of BAs underpin their distinct signaling, metabolic, and immunological functions.

In human hepatocytes, cholesterol 7α‐hydroxylase (CYP7A1) catalyzes the conversion of cholesterol into the primary BAs cholic acid (CA) and chenodeoxycholic acid (CDCA) [4] (Figure 1A). Before secretion from the liver across the canalicular membrane, CA and CDCA are conjugated at C‐24 with glycine or taurine by BA‐CoA synthase (BACS) and BA‐amino acid transferase (BAT), generating glyco‐ and tauro‐derivatives [11] (Figure 1A). This conjugation lowers their pKa to approximately 5, resulting in complete ionization of conjugated BAs (CBAs), which are therefore referred to as bile salts. These bile salts are stored in the gallbladder together with phosphatidylcholine and cholesterol [12]. Upon cholecystokinin‐induced gallbladder contraction, approximately 20–30 g/day of BAs are released into the duodenum, where CBAs activate pancreatic lipase and form mixed micelles with cholesterol, partially ionized fatty acids, and fat‐soluble vitamins (A, D, K, and E), thereby facilitating their absorption across the intestinal epithelium [13]. Approximately 95% of BAs are reabsorbed in the intestine and recycled through the enterohepatic circulation [14]. Unconjugated BAs, and to a lesser extent glycine‐conjugated BAs, are passively reabsorbed along the small intestine, whereas CBAs are actively reabsorbed in the distal ileum via the ileal BA transporter (IBAT). Subsequently, BAs are secreted into the portal circulation by the heterodimeric organic solute transporter OSTα/OSTβ. Hepatic uptake is mediated by the sodium‐dependent taurocholate co‐transporting polypeptide (NTCP/SLC10A1) and organic‐anion‐transporting polypeptides (OATPs). Notably, intracellular BA levels regulate their own synthesis through tightly controlled feedback mechanism [15, 16, 17].

### 2.2 Gut Microbiota Metabolism of BAs

A fraction of primary BAs escapes enterohepatic circulation and reaches the colon, where the GM converts them into secondary BAs. These microbial transformations diversify the BA pool and increase its overall hydrophobicity, thereby enhancing the passive reabsorption of secondary BAs [18]. Specifically, BA metabolism by the GM involves several key enzymatic reactions. The initial and most prevalent step is deconjugation, catalyzed by bile salt hydrolases (BSHs), which belong to the N‐terminal nucleophilic hydrolase (Ntn) family and hydrolyze the C‐24 N‐acyl bond, releasing glycine or taurine from CBAs [19].

BSH activity is widespread among gram‐positive bacterial genera, including *Clostridium*, *Enterococcus*, *Bifidobacterium*, and *Lactobacillus*, with BSH genes identified in species such as *Clostridium perfringens* , *Lactobacillus plantarum* , *Lactobacillus johnsonii* , *Bifidobacterium longum* , *Bifidobacterium bifidum* and *Listeria monocytogenes* [20, 21, 22, 23, 24]. Among gram‐negative bacteria, BSH expression has been reported primarily in *Bacteroides* species [25]. Notably, rodent studies have shown that BA consumption at levels comparable to those associated with high‐fat diets reduces the abundance of Bacteroidetes while promoting the growth of Firmicutes [26].

Beyond providing amino acids for bacterial metabolism, BA deconjugation significantly influences GM composition. Due to their detergent‐like properties, BAs exert antimicrobial effects by disrupting membrane integrity, even at low concentrations. Deconjugation of primary BAs increases their lipophilicity, enhancing interaction with membrane lipids and facilitating passive flip‐flop across phospholipid bilayers [27]. In addition, primary CA and CDCA undergo multistep 7α‐dehydroxylation (originally characterized in *Clostridium scindens* ) to produce the secondary BAs deoxycholic acid (DCA) and lithocholic acid (LCA), respectively [28] (Figure 1B).

Further bacterial enzymatic modifications, including oxidation, epimerization, desulfation, esterification, and conjugation, contribute to the extensive structural diversity of the BA pool [29]. For instance, in humans, bacterial 7β‐hydroxysteroid dehydrogenase catalyzes the conversion of small amounts of CDCA into ursodeoxycholic acid (UDCA) [30] (Figure 1B). Recent studies also indicate that esterified forms of primary BAs constitute a substantial fraction of the human fecal BA pool. Moreover, emerging evidence suggests that bacteria are capable not only of deconjugating but also of conjugating amino acids to the C‐24 carbonyl group of BAs. A metabolomic comparison of germ‐free and conventional mice identified GM‐dependent conjugation of CA with phenylalanine, tyrosine, and leucine [31]. Consistently, in vitro studies have demonstrated that several bacterial species, particularly within the *Bifidobacterium*, *Bacteroides*, and *Enterococcus* genera, can conjugate amino acids to BAs [32].

Collectively, these microbial transformations markedly increase the structural complexity and functional repertoire of the BA pool, with important implications for host physiology and disease pathogenesis, particularly when these processes become dysregulated.

## 3 BAs as Key Signaling Molecules in the GM–Immunity Axis

Building on the overview of BA metabolism and host–microbiota interactions described above, this section focuses on the role of BAs as signaling molecules within the GM–immunity axis. Rather than acting solely as digestive detergents, BAs engage a defined set of host receptors to modulate metabolic and immune pathways. Understanding these core signaling mechanisms is essential for interpreting how alterations in BA composition are linked to inflammatory and neurological diseases, which are discussed in subsequent sections.

**FIGURE 2:**
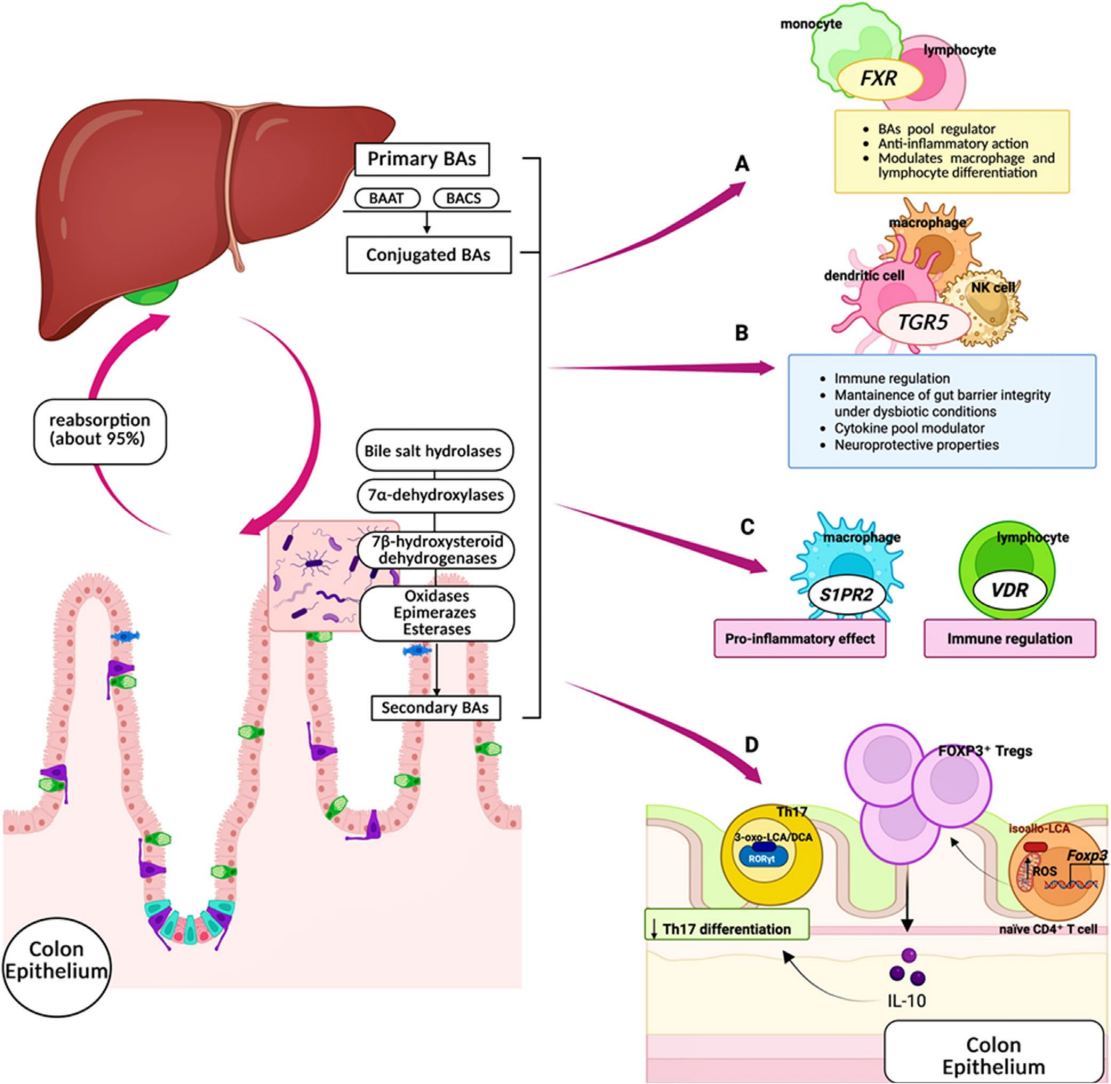
Primary BAs, synthesized in the liver and conjugated to glycine or taurine prior to secretion, are transformed by GM into secondary BAs which engage distinct receptors on immune cells to shape gut immunity. (A) FXR expressed on monocytes and lymphocytes regulates BA homeostasis and exerts anti‐inflammatory effects. (B) TGR5 found on macrophages, dendritic cells, and NK cells contributes to immune regulation, maintenance of gut barrier integrity, and cytokine modulation. (C) S1PR2 expressed on macrophages drives pro‐inflammatory NF‐κB signaling while VDR expressed on lymphocytes induces anti‐inflammatory gene transcription and upregulates detoxification pathways. (D) T‐Cell Modulation by BA derivatives: 3‐oxo‐LCA/DCA bind RORγt to inhibit Th17 differentiation while isoallo‐LCA enhances mitochondrial ROS to upregulate FOXP3 transcription, expanding Tregs and fostering IL‐10‐mediated immune tolerance. Through distinct receptor pathways on immune cells, secondary bile acids regulate both T cell differentiation and innate immune signaling to maintain intestinal homeostasis. *Source:* Created with BioRender.com.

BAs are not only essential for the digestion and absorption of dietary fats but also act as potent signaling molecules that modulate numerous physiological processes, particularly inflammation. By engaging receptors such as FXR and TGR5, BAs regulate metabolic pathways and immune responses [33] (Figure 2). Inflammation is highly context‐dependent and can be either protective or detrimental; similarly, BAs exert dual effects on inflammatory cascades. While necessary for lipid emulsification, excessive BA accumulation, as observed in IBD, can exacerbate tissue damage [34]. Although the precise causal links between BA–receptor activation and immunomodulatory effects remain under active investigation, recent studies are beginning to map how distinct BAs selectively activate FXR, TGR5, and other receptors to elicit pro‐ or anti‐inflammatory outcomes [35]. Moreover, interactions between BAs and the GM add another layer of complexity: microbially derived secondary BAs often exhibit altered receptor affinities and bioactivities, offering promising avenues for therapies targeting BA‐driven signaling pathways [36].

### 3.1 Farnesoid X Receptor

FXR is a nuclear receptor expressed in multiple cell types, including immune cells such as monocytes and lymphocytes, albeit at levels approximately tenfold lower than in hepatocytes [37]. As a master regulator of BA homeostasis, FXR activation exerts potent anti‐inflammatory effects [38] (Figure 2A). A key mechanism involves the transcriptional regulation of genes controlling BAs synthesis, conjugation, and transport, thereby preventing BA overload and subsequent pro‐inflammatory signaling [38]. Beyond metabolic regulation, FXR directly influences immune responses by modulating macrophage and lymphocyte differentiation. For example, FXR activation is associated with increased regulatory T cells (Tregs) differentiation and may contribute to immune tolerance [39]. A 2024 study demonstrated that FXR signaling is disrupted following inflammation‐induced epithelial injury in a murine model of colitis‐associated colorectal cancer. In this context, aberrant BA signaling is associated with macrophage–intrinsic FXR, leading to pro‐inflammatory cytokine secretion and enhanced intestinal stem‐cell proliferation [40]. FXR also functions as a negative regulator of NF‐κB–mediated hepatic inflammation by suppressing pro‐inflammatory mediators (e.g., iNOS, COX‐2, interferon‐inducible protein 10, and interferon‐γ) while preserving or enhancing NF‐κB–driven anti‐apoptotic pathways. Conversely, NF‐κB activation can inhibit FXR‐dependent gene transcription, highlighting a bidirectional negative crosstalk [41].

Consinstent with these mechanisms, obeticholic acid (OCA), a potent FXR agonist, exhibits therapeutic activity across several liver and intestinal disorders. In nonalcoholic fatty liver disease (NAFLD), OCA reduces hepatic lipid accumulation and improves metabolic regulation [42]; in metabolic‐associated steatohepatitis, it suppresses inflammatory and fibrogenic pathways [43].

In cholestatic diseases, FXR activation by OCA normalizes BA synthesis and detoxification, limiting hepatocellular injury [44]. In the intestine, OCA enhances mucosal barrier integrity and attenuates inflammation, providing protection in experimental colitis models [38]. Additionally, FXR activation is associated with reduced markers of hepatic inflammation and preservation of tissue integrity in experimental models [45].

### 3.2 Takeda G Protein‐Coupled Receptor 5

TGR5, also known as G protein‐coupled bile acid receptor 1 (GPBAR1), is highly expressed in macrophages, monocytes, dendritic cells, natural killer (NK) cells and cholangiocytes [46, 47]. Its endogenous ligands, ranked by agonist potency, are LCA, DCA, CDCA, CA and UDCA. Activation of TGR5 by both primary and secondary BAs is linked to modulation of immune responses [48]. In intestinal epithelial cells, TGR5 stimulation is associated with glucagon‐like peptide‐1 (GLP‐1) secretion, a hormone with anti‐inflammatory properties that preserves gut barrier integrity and mitigates immune dysregulation in IBD [49] (Figure 2B). Consistently, a 2020 study reported that germ‐free mice exhibit disrupted postprandial BA profiles and impaired GLP‐1 responses, whereas fecal microbiota transplantation (FMT) restores both [50]. TGR5 also modulates cytokine production, influencing the nature and magnitude of immune responses in the gut and liver [2, 51]. Given its dual role in immune and metabolic regulation, TGR5 represents an attractive therapeutic target for metabolic disorders and diseases associated with gut dysbiosis and inflammation.

### 3.3 Other Pathological Signaling Pathways

Additional BA receptors, including sphingosine‐1‐phosphate receptor 2 (S1PR2), the vitamin D receptor (VDR), and the pregnane X receptor (PXR), are primarily engaged under pathological conditions characterized by elevated BA levels. S1PR2, expressed in macrophages and liver sinusoidal endothelial cells, is preferentially activated by CBAs [52, 53]. Upon stimulation, S1PR2 promotes cholangiocyte proliferation and triggers pro‐inflammatory signaling via NF‐κB, contributing to liver damage; it also modulates immune cell trafficking, amplifying tissue inflammation [54, 55, 56] (Figure 2C). In contrast, VDR is predominantly expressed in immune cells, including B cells, T cells, T regs, monocytes, dendritic cells, and to a lesser extent, macrophages [57]. BA‐mediated VDR activation induces transcription of anti‐inflammatory genes while suppressing pro‐inflammatory pathways [58, 59] (Figure 2C). VDR also upregulates detoxification enzymes, such as cytochrome P450, enhancing BA clearance and protecting hepatic and intestinal tissues from BA‐induced toxicity [60]. This receptor‐ligand interaction is highly relevant to diseases such NAFLD and IBD. Finally, PXR activation enhances transcription of genes involved in detoxifying inflammatory mediators and xenobiotics, conferring protective effects against immune dysregulation [61, 62].

## 4 Disease Applications of BAs

Having outlined the principal BA‐activated signaling pathways involved in immune regulation, this section examines how disruptions of the BA–GM–immunity axis are associated with human diseases. Rather than implying direct causality, the following examples illustrate how disease‐specific alterations in BA composition and signaling are linked to inflammatory and neurological phenotypes, highlighting both translational opportunities and current limitations.

BA‐mediated pathways have recently emerged as compelling therapeutic targets for a broad spectrum of diseases, including IBD and neurological disorders. In parallel, detailed characterization of BA structural features that determine receptor potency and selectivity has driven the development of next‐generation BA‐based therapeutics. Notably, several natural and semi‐synthetic derivatives, such as OCA and norUDCA, have already demonstrated clinical benefits in metabolic diseases. Collectively, as summarized in Table 1, these advances highlight the growing relevance of BAs in disease pathophysiology and underscore their expanding potential as therapeutic agents.

**TABLE 1:** BAs and BA‐derived compounds used as treatment drugs for intestinal and neurological diseases.

Target/pathway	BA or BA‐derived compound	Proposed mechanism/principal immune effect	Disease	References
RORγt	3‐oxoLCA/DCA	Inhibition of Th17 differentiation and associated cytokine production	CD	[63]
NR4A1	Isoallo‐LCA	Modulation of T‐cell activation and inflammatory gene expression	CD	[64]
TGR5	LCA/DCA	Modulation of macrophage and innate immune responses	CD	[65]
TGR5/FXR	TUDCA	Modulation of stress‐response and inflammatory pathways	ALS	[66]
TGR5/FXR	UDCA	Altered immune signaling and epithelial barrier function	CD	[67]
TGR5/FXR/PXR	TUDCA	Attenuation of inflammatory responses	CD	[68]
GM	GUDCA	Reduction of inflammatory cytokines expression	CD	[69]
GM	TUDCA	Reduced inflammatory cytokine levels and improved gut barrier function	NAFLD	[70]
FXR	OCA	Reduced intestinal inflammation and improved BA homeostasis	UC	[38]
FXR	UDCA	Anti‐inflammatory signaling in colonic epithelium	UC	[71]
FXR	OCA	Improved lipid metabolism and reduced hepatic inflammation	NAFLD	[42]
FXR	HDCA	Altered lipid and glucose metabolism	NAFLD	[72]
FXR	OCA	Improved cholestatic markers	Cholestasis	[44]
PKC‐α	TUDCA	Cytoprotective and anti‐apoptotic signaling	Cholestasis	[73]
PPAR‐γ	NorUDCA	Anti‐inflammatory metabolic effects	NAFLD	[74]
TMEM16A	UDCA	Modulation of epithelial ion transport and barrier integrity	Cholestasis	[75]
VDR	LCA	Regulation of mucosal immune responses	UC	[76]
γ‐secretase	TUDCA	Inhibition of amyloidogenic processing associated with neuroprotective effects	AD	[77]

### 4.1 Altered BA–GM–Immunity Axis in IBD

IBD represents one of the most extensively studied contexts in which perturbations of the BA–GM–immunity axis have been linked to chronic inflammation. Disruption of the balance between pro‐inflammatory T helper 17 (Th17) cells and Tregs, a process influenced by alterations in BA signaling pathways described above, is a hallmark of chronic inflammatory disorders such as IBD. This interplay is finely tuned by both host‐ and microbe‐derived BAs [78] (Figure 2D). In the colonic mucosa, approximately two‐thirds of Tregs co‐express the Th17 master regulator RORγt and restrain Th17 differentiation via IL‐10 secretion [79]. This regulatory network is further modulated by secondary BA derivatives, including 3‐oxo‐LCA, 3‐oxo‐DCA and isoallo‐LCA (Figure 1B). Consistent with the immunomodulatory mechanisms outlined in Section 3, secondary BA derivatives such as 3‐oxo‐LCA, 3‐oxo‐DCA, and isoallo‐LCA have been associated with modulation of Th17/Treg differentiation in experimental and human IBD settings [79, 80]. In IBD patients, dysbiosis, characterized by elevated primary BAs and depleted secondary BAs, is associated with isoallo‐LCA levels and diminished abundance of microbial genes involved in its biosynthesis [81]. Sixteen bacterial species across eleven genera, including *Bacteroides*, *Bifidobacterium*, *Lactobacillus* and *Parabacteroides*, can convert 3‐oxo‐LCA into isoallo‐LCA, underscoring the potential of restoring microbial BA metabolism as a therapeutic strategy [82]. Beyond immunomodulation, BA profiling has emerged as a dynamic biomarker strategy. In active CD, lower secondary BA levels distinguish flares from remission [83], whereas in UC patients with ileal pouch–anal anastomosis, loss of secondary BA–producing bacteria correlates with DCA/LCA depletion [84]. Additionally, UDCA supplementation has been associated with reduced inflammation in preclinical colitis models [51]. Active IBD is also associated with increased total fecal BAs, driven by primary BAs, reduced secondary BAs, and elevated 3‐hydroxy metabolites [83]. Quinn et al. recently identified novel microbially derived BAs in IBD stool samples, namely leucocholic, phenylalanocholic and tyrosocholic acids, that act as potent FXR agonists, implicating them in disease pathogenesis [31]. Serum BA profiles further distinguish healthy individuals from IBD patients based on UDCA, tauro‐UDCA (TUDCA) and glyco‐UDCA (GUDCA) levels (Figure 1C) [69]. In CD patients, anti‐TNFα therapy is associated with higher total serum BAs and increased secondary BAs compared to untreated patients [85]. Clinically, enrichment of secondary glyco‐LCA (GLCA) and glyco‐DCA (GDCA) acids (Figure 1C), or detection of microbial bile acid‐inducible (*bai*) genes, has been associated with early remission under anti‐cytokine therapy [86]. In pediatric CD, exclusive enteral nutrition responders exhibit rising LCA levels [87], and FMT in IBD complicated by *Clostridioides difficile* shifts BA profiles toward donor‐like secondary BA enrichment [88]. Collectively, these findings underscore the potential of BA profiling as both a diagnostic and therapeutic guide in IBD, enabling monitoring of disease activity, prediction of therapeutic outcomes, and management of complications.

### 4.2 Altered BA–GM–Immunity Axis in Neurological Disorders

Beyond intestinal inflammation, accumulating evidence suggests that BA signaling also influences extra‐intestinal organs. In particular, the ability of certain BAs to reach the central nervous system (CNS) and engage local receptors has prompted investigation of their potential role in neurological disorders.

BAs form a chemically diverse pool in the brain, including primary and secondary, conjugated and unconjugated species, whose lipophilicity allows them to cross the blood–brain barrier via both passive diffusion and active transport. Once in the CNS, BAs engage locally expressed receptors, establishing a gut–brain signaling axis complementary to vagal and endocrine pathways [89]. Additionally, primary BAs can be synthesized in situ via the alternative pathway mediated by CYP27A1, CYP8B1, and CYP46A1 [90]. Extending the BA signaling mechanisms described in Section 3 to the CNS, emerging evidence suggests that alterations in FXR signaling are associated with changes in neurotransmitter profiles and neurobehavioral outcomes in experimental models. Mice lacking FXR exhibit altered neurotransmitter profiles in the hippocampus and cerebellum, accompanied by cognitive and motor deficits [91]. In line with the immunometabolic functions of TGR5 signaling discussed above, BA–TGR5 interactions have also been linked to gut–brain communication through both peripheral endocrine pathways and central immune modulation [92]. In vitro, the TGR5 agonist betulinic acid reduces lipopolysaccharide‐induced inflammatory and phagocytic responses in microglia, suggesting anti‐inflammatory potential within the CNS [93]. Several BAs have shown neuroprotective properties in models of neurodegeneration. TUDCA attenuates amyloid‐β‐induced neuronal apoptosis in vitro and reduces amyloid plaque burden while improving behavior in APP/PS1 Alzheimer's mice [77, 94]. CDCA administration likewise decreases extracellular Aβ deposition and neuronal loss, yielding functional benefits [95]. In Parkinson's disease models, UDCA and TUDCA mitigate dopaminergic neuron degeneration [96]. In amyotrophic lateral sclerosis, TUDCA improves motor performance and extends survival in both preclinical and clinical studies [97]. Interestingly, serum profiling in ALS patients revealed elevated, rather than deficient, TUDCA levels, indicating that factors such as diet or GM alterations may modulate its concentration [98].

Together, these findings position BAs as key gut‐derived neuromodulators and potential therapeutics for CNS disorders, although causal links between endogenous BA dysregulation and neurodegeneration remain to be fully established.

### 4.3 Challenges in Translating BA Biomarkers Into Clinical Practice

Despite the growing therapeutic promise of BA‐mediated pathways, pronounced interindividual variability in BA homeostasis presents a critical challenge for both diagnostics and therapeutics. The BA pool composition is influenced by demographic, metabolic, and microbial factors, including sex‐ and ethnicity‐related differences. In healthy individuals, total BA synthesis can vary more than ninefold and is approximately 29% higher in men than women [99]. Ethnicity also contributes to variability: Asian populations exhibit significantly higher circulating levels of CDCA, glyco‐CDCA, tauro‐CDCA and glyco‐CA compared with Caucasians [100]. These differences may reflect reduced microbial BSH and dehydrogenase activities and altered abundances of key bacterial taxa. For example, South Asian UC patients show lower relative abundances of *Ruminococcus* and *Anaerostipes*, genera implicated in anti‐inflammatory secondary BA production, and higher levels of *Bifidobacterium*, *Lactobacillus*, and *Streptococcus* compared with Caucasians [101]. Ethnicity‐specific variations in host gene expression have also been repoted; a 2021 study found elevated FXR expression in Hispanics relative to African Americans and Caucasians [102].

Species‐specific differences further limit translational relevance.

It is important to note that muricholic acids (α‐, β‐, and ω‐MCA) are synthesized in rodents but are absent in humans. These species‐specific BAs exhibit distinct affinities for FXR and TGR5, compared with human primary and secondary BAs. Consequently, murine models may not fully recapitulate human BA signaling dynamics or immunomodulatory effects, limiting direct translation of mechanistic findings [103, 104].

Divergent GM compositions, especially at deep taxonomic levels, complicate extrapolation: genera such as *Prevotella* and *Faecalibacterium*, the latter a well‐established marker of anti‐inflammatory microbiota in IBD in remission, occur at substantially lower abundances in mice [105, 106, 107, 108].

Differences in immune system architecture, cytokine signaling, T‐cell polarization, and tolerance mechanisms, combined with anatomical variations (gut length, surface area, and luminal pH gradients), further constrain direct translation [109, 110].

Even more human‐like models, such as pigs, maintain microbial communities distinct from humans [111]. Experimental approaches such as germ‐free or antibiotic‐treated animals create artificially simplified microbiomes that fails to recapitulate chronic, multifactorial human diseases like IBD [112, 113, 114]. To overcome these limitations, emerging platforms aim to more faithfully replicate human physiology, including gnotobiotic mice colonized with human GM, genetically engineered models expressing human BA transporters or receptors, and organ‐on‐chip or bioengineered gut systems integrating human cells in controlled microenvironments [115, 116]. These innovative models provide opportunities to dissect BA–GM–immune interactions with higher fidelity, enhancing mechanistic insights and translational potential for human health.

### 4.4 From Mechanism to Clinical Application: How BA Signatures Inform Disease Management

While mechanistic studies have elucidated how BAs signal through receptors such as FXR and TGR5 to modulate immune responses, clinical translation requires an integrative framework linking these pathways to measurable disease‐associated BA patterns. Rather than acting as isolated mediators, BAs represent molecular interfaces that reflect upstream host–microbial interactions and downstream immune phenotypes. In IBD, for example, alterations in secondary BA abundance are associated with shifts in regulatory versus pro‐inflammatory immune signatures and correlate with disease activity states such as remission or flare. Similarly, in neurological disorders, changes in circulating or brain‐accessible BA species observed in preclinical and clinical studies may reflect altered neuroimmune signaling rather than direct pathogenic drivers.

Viewed through this lens, BA profiling serves as a translational bridge between mechanistic insights and clinical observation, supporting patient stratification and hypothesis‐driven therapeutic targeting, while underscoring the need for prospective validation.

## 5 Analytical Advances in BA Profiling

Accurate and comprehensive characterization of BA profiles in biological samples is essential to translate the mechanistic and associative insights described in Sections 3 and 4 into clinically meaningful biomarkers and therapeutic strategies.

To support diagnostic and therapeutic applications, a range of analytical techniques has been developed for sensitive and comprehensive qualitative and quantitative measurement of BAs in human samples. HPLC–MS/MS has emerged as the gold standard for BA profiling, while GC–MS remains valuable for specific applications. Other approaches, including conventional LC–MS, immunoassays, and nuclear magnetic resonance (NMR) spectroscopy, currently play more limited roles [117] (Table 2).

**TABLE 2:** Comparison of major analytical platforms for BA profiling. This table summarizes key features, strengths, and limitations of the most commonly used analytical methods, including GC–MS, HPLC–MS/MS, UPLC–MS/MS, NMR, and immunoassays.

Platform	Strengths	Limitations	Typical applications
GC–MS	High sensitivity, excellent separation of derivatized compounds	Requires derivatization; labor‐intensive; limited stereoisomer resolution	Research; identification of niche BA metabolites
HPLC–MS/MS	Broad BA coverage, robust quantification, good sensitivity	Moderate throughput; sample cleanup required; matrix effects	Translational research using plasma feces and bile samples
UPLC–MS/MS	Ultra‐high resolution; rapid analysis; low detection limits	Expensive instrumentation; complex method optimization	High‐throughput research; multi‐BA quantification
NMR	Structural information; no derivatization	Low sensitivity; high sample concentrations required	Structural characterization; metabolomics studies
Immunoassays	Simple, rapid, high‐throughput	Limited specificity; cross‐reactivity; often measures total or selected BAs	Screening; routine clinical studies

### 5.1 GC/MS

GC–MS was among the earliest and most sensitive methods applied to BA analysis [118]. However, the low volatility and thermal lability of BAs, attributable to their carboxyl and hydroxyl functional groups, necessitate extensive sample preparation and derivatization. Typically, this workflow begins with chemical (e.g., alkaline hydrolysis) or enzymatic hydrolysis (e.g., using choloylglycine hydrolase or β‐glucuronidase), followed by derivatization to enhance volatility. Carboxyl groups are most commonly methylated using reagents such as methanolic HCl, methanol‐sulfuric acid, or p‐toluenesulfonic acid, whereas hydroxyl groups are converted into trimethylsilyl ethers using reagents such as N,O‐bis(trimethylsilyl)acetamide (BSA) or bis(trimethylsilyl)trifluoroacetamide (BSTFA). Quantitative accuracy relies on stable isotope‐labeled internal standards to correct for variability introduced during sample preparation and injection [119]. Despite its pioneering role and high intrinsic sensitivity, GC–MS is limited by time‐consuming workflows and suboptimal resolution of stereoisomers. Consequently, it has largely been supplanted by more efficient platforms such as HPLC–MS. Nevertheless, ongoing advances in automated sample preparation and high‐temperature capillary columns may reestablish GC–MS for niche applications requiring exceptional chromatographic resolution.

### 5.2 HPLC–MS/MS

HPLC is widely used for BA analysis because of its high sensitivity and resolving power in complex biological matrices, including serum, bile, intestinal biopsies and feces [119]. A typical workflow begins with sample pretreatment (e.g., protein precipitation, lipid removal and desalting), followed by separation on a reversed‐phase C18 silica column. Mobile phases commonly consist of acetonitrile, ammonium formate, and formic acid. Individual BAs elute sequentially and have historically been detected by ultraviolet (UV) spectrometry. However, although HPLC–UV can quantify BAs at micromolar to millimolar concentrations, the inherently weak UV absorbance of most BAs limits sensitivity below the submicromolar range, necessitating pre‐column derivatization [120].

Several derivatization strategies have been developed to overcome this limitation. In 2015, Shi et al. demonstrated that derivatization with 2‐bromo‐4′‐nitroacetophenone, catalyzed by a crown ether, produces derivatives absorbing at 263 nm, substantially lowering detection limits [121]. Alternatively, phenacyl bromide has been used to derivatize both primary and secondary BAs, including stereoisomers such as isoLCA and isoDCA, allowing detection at 253 nm [10].

**FIGURE 3:**
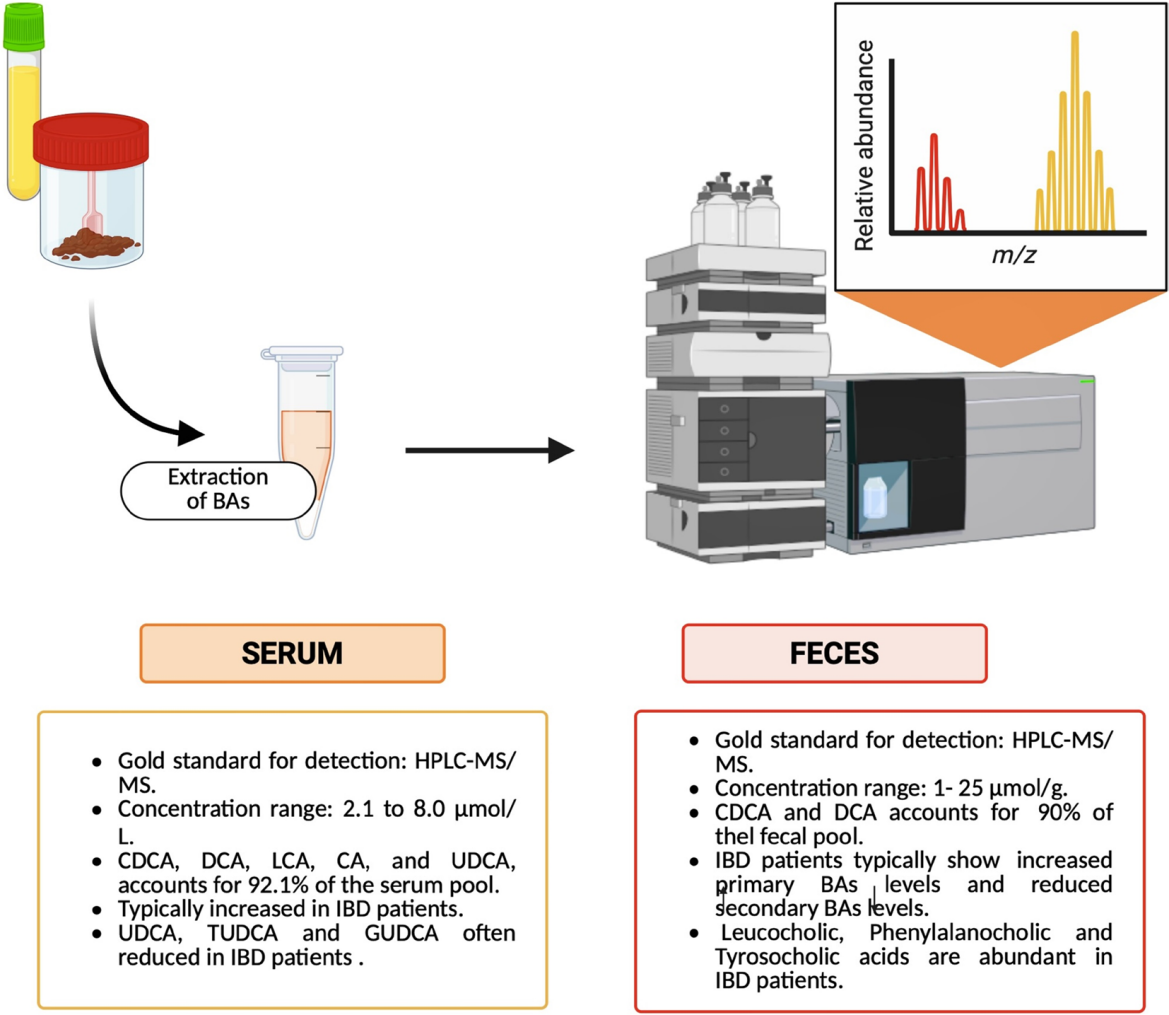
Gold standard HPLC–MS/MS method for BAs quantitative and qualitative analysis from serum and fecal samples. This method enables precise profiling of BA composition in biological samples, supporting mechanistic and clinical studies. *Source:* Created with BioRender.com.

Despite these advances, HPLC–UV workflows remain labor‐intensive and time‐consuming. To streamline analysis and enhance sensitivity, HPLC–MS/MS has become the gold standard for simultaneous detection of conjugated and unconjugated BAs (Figure 3) [122]. Negative‐mode electrospray ionization (ESI) efficiently ionizes BAs via their carboxyl groups. Careful optimization of mobile‐phase pH is critical, as it affects BA ionization efficiency, chromatographic retention and MS signal intensity. Accurate quantification further depends on rigorous sample cleanup to remove proteins, lipids and salts, as well as the use of deuterated internal standards to correct for variability in extraction efficiency and instrument response [123].

In human feces, BA concentrations typically range from 1 to 25 μmol/g dry weight due to efficient enterohepatic circulation and are most reliably quantified by HPLC–MS/MS [124, 125]. In adults, unconjugated secondary BAs, predominantly DCA and LCA, constitute up to 90% of the total fecal BA pool, whereas in meconium and infant feces, primary BAs can account for up to 50% of the total [126, 127]. Zheng et al. recently reported an HPLC–MS/MS method for simultaneous quantification of conjugated and unconjugated BAs in mouse feces, confirming DCA and CDCA as the most abundant metabolites [128].

In serum, total BA concentrations in healthy adults typically range from approximately 2.1 to 8.0 μmol/L, with CDCA (irrespective of conjugation status) representing the predominant species [129, 130]. Together with DCA, LCA, CA, and UDCA, CDCA accounts for approximately 92.1% of the total BA pool. Using HPLC–MS/MS, Bathena et al. reported a mean total serum BA concentration of 3.89 μM in healthy individuals, of which 33% were sulfated, 55% glycine‐conjugated, and 13% taurine‐conjugated [129].

The advent of ultra‐performance liquid chromatography (UPLC)–MS/MS has further advanced BA profiling. Improvements in column technology, including sub‐2 μm particles packing and increased pressure tolerance (up to 1300 bar), have enabled faster and higher‐resolution separations. In 2015, Han et al. described a UPLC–MS method capable of resolving 50 distinct BAs in human serum at sub‐nanomolar concentrations, surpassing the sensitivity of earlier HPLC–MS/MS approaches [131]. More recently, a rapid UPLC–MS protocol employing salting‐out‐assisted liquid–liquid extraction enabled BA quantification within 4 min and 32 s [132]. Similarly, Ramos‐Garcia et al. used UPLC–MS/MS to quantify 28 BAs and six sulfated derivatives in murine feces, identifying DCA, α‐MCA and β‐MCA acids as the most abundant species, and glycocholic acid sulfate as the least abundant [133]. Collectively, these technological advances have markedly improved the throughput, sensitivity, and robustness of BA analysis in both research and clinical settings.

### 5.3 Pre‐Analytical and Matrix Considerations

Several pre‐analytical and matrix‐related factors can influence BA measurements. Diet, circadian rhythms, and recent medication intake may alter circulating and fecal BA profiles. Sample handling, including collection, storage temperature, and freeze–thaw cycles, can affect the stability of conjugated and unconjugated BA species. Moreover, differences between biological matrices, such as serum versus feces, can introduce variable ion suppression or matrix effects, potentially impacting the sensitivity and accuracy of MS‐based assays. Careful standardization of collection, storage, and processing procedures is therefore essential for reproducible and clinically meaningful BA profiling [134]. Despite advances in analytical technologies, several critical gaps remain before BA profiling can be fully integrated into clinical practice. These include a lack of standardized protocols for sample collection, storage, and preparation; inter‐laboratory variability in extraction efficiency and instrument calibration; and incomplete coverage of BA species, particularly rare or microbially conjugated metabolites. Furthermore, differences in matrix composition and potential ion suppression in complex biological samples can affect reproducibility. Addressing these challenges is essential for establishing reliable BA‐based biomarkers and ensuring comparability of results across studies and centers.

## 6 Conclusions and Future Perspectives

**FIGURE 4:**
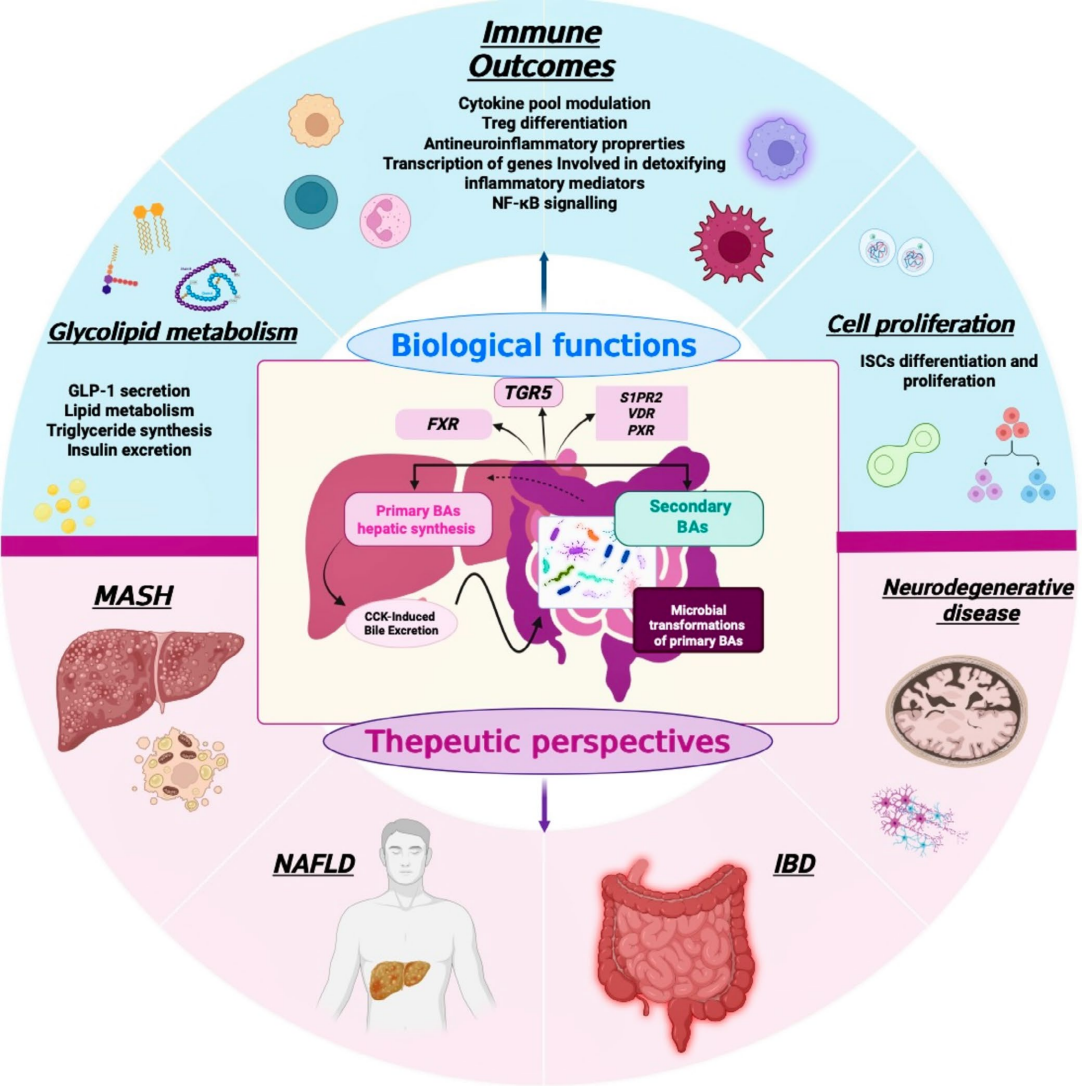
Overview of BAs biology and their therapeutic relevance. Primary BAs synthesized in the liver undergo microbial transformations in the gut to form secondary BAs, which signal through receptors such as FXR, TGR5, S1PR2, VDR, and PXR. These signaling pathways modulate diverse biological functions, including immune responses, glycolipid metabolism, and cell proliferation. Dysregulation of BA pathways is implicated in MASH, NAFLD, IBD, and neurodegenerative disorders, highlighting BAs as promising therapeutic targets. Overall, modulating BA pathways offers potential for treating metabolic, inflammatory, and neurodegenerative diseases.

Although further studies are required to fully elucidate the cellular and molecular mechanisms by which BAs regulate immune responses, their pivotal roles in a wide range of physiological and pathological processes are increasingly evident. Given the complex and dynamic interplay between BAs and the GM, advancing our understanding of this multifaceted axis may open new avenues for the management of chronic inflammatory, metabolic, and neurodegenerative diseases. Indeed, the identification and functional characterization of distinct BA species and their cognate receptors have already revealed promising therapeutic targets (Figure 4).

In particular, growing evidence that microbially derived secondary BAs can selectively modulate immune responses highlights the potential to harness these metabolites for the development of functional foods, probiotics, and targeted nutraceutical interventions. Moreover, while recent advances in analytical technologies have substantially improved the precision and sensitivity of BA profiling, the establishment of standardized and validated protocols for measuring BAs, especially in human serum and fecal samples, remains essential to fully realize their potential as reliable, non‐invasive biomarkers of gut dysbiosis and related diseases.

Despite these advances, several important limitations must be acknowledged. Human BA profiles are highly heterogeneous and are influenced by factors such as diet, genetics, GM composition, age, sex, and ethnicity, which can complicate the interpretation of observational data. In addition, most mechanistic insights are derived from animal models, particularly mice, which exhibit distinct BA metabolism, GM composition, and immune system architecture compared with humans. Consequently, translating findings from preclinical studies to human disease remains challenging. Furthermore, few prospective studies have validated BAs as predictive biomarkers, and current BA‐omics pipelines lack standardized protocols across laboratories, thereby limiting the reproducibility and comparability of results. Addressing these gaps will be crucial for the development of reliable diagnostic tools and personalized therapeutic strategies.

In conclusion, deeper insight into BA signaling pathways, interindividual variability in BA pool composition, and BA–GM interactions will be critical for the development of personalized therapies and innovative diagnostics. Realizing this potential will require rigorous validation in standardized, prospective clinical cohorts, combined with harmonized analytical pipelines and careful consideration of interspecies differences.

## Author Contributions

S.B. and M.T. designed the study and wrote the manuscript; F.C. and M.M. acquired relevant data; G.B. and A.A. contributed to the critical revision of the manuscript. All authors have read and agreed to the published version of the manuscript.

## Funding

This work was supported by Microbiome‐immunity axis: functional food for the inflammation modulating in gastrointestinal diseases “FONZIE”, a project funded by University as a “problem‐driven” research project to be carried out through the establishment of public‐private partnerships in the context of the issues of the PNR (ex D.M. 737/2021). The call is part of the initiatives financed by the European Union—Next Generation EU, CUP: B83C22003920001.

## Conflicts of Interest

The authors declare no conflicts of interest.

## Data Availability

Data sharing not applicable to this article as no datasets were generated or analysed during the current study.
